# Postoperative Fracture Risk in Giant Cell Tumor: A Case Report and Review of Literature

**DOI:** 10.7759/cureus.46192

**Published:** 2023-09-29

**Authors:** Amit Kumar, Kumar Keshav, Siddhartha Singh, Amarendra Singh

**Affiliations:** 1 Orthopaedics, Sanjay Gandhi Postgraduate Institute of Medical Sciences, Lucknow, IND; 2 Trauma and Orthopaedics, Sanjay Gandhi Postgraduate Institute of Medical Sciences, Lucknow, IND

**Keywords:** defect size, bisphosphonates, proximal femur, recurrence, gct

## Abstract

Giant cell tumor (GCT) of the proximal femur poses various challenges in its management and recurrence. We present a rare case of GCT of proximal femur in which recurrence and coxa vara deformity were encountered after index surgery. Management of the recurrence was done with intramedullary fixation with extended curettage and bone grafting. Different aspects of management such as the role of defect size, adjuvants, bone cement/bone graft, implants, and bisphosphonates have been highlighted in this article.

## Introduction

Giant cell tumors of the bone (GCTB) are intermediate-grade primary bone tumors [1]. They are mostly treated by intralesional curettage to maintain joint integrity and functional outcome. Post curettage, the defect that is created can be filled with bone cement, bone autograft/allograft, or bone graft substitutes. However, there is still an imminent risk (up to 14%) of fracture in the postoperative period [2-4].

Giant cell tumor (GCT) of the proximal femur (PF) accounts for around 6% of all GCTs [5]. The treatment of a GCT around the PF is unclear. In most cases, the management relies on the surgeon’s experience and the patient’s age [6]. Various treatment modalities have been used depending on the defect size created by the tumor. However, there is still a paucity of literature on the treatment of GCT in the PF.

In this case report, we describe a rare case of GCT of the PF where the patient developed post-surgery deformity and fracture and how we managed it with a review of the literature.

## Case presentation

**Figure 1:**
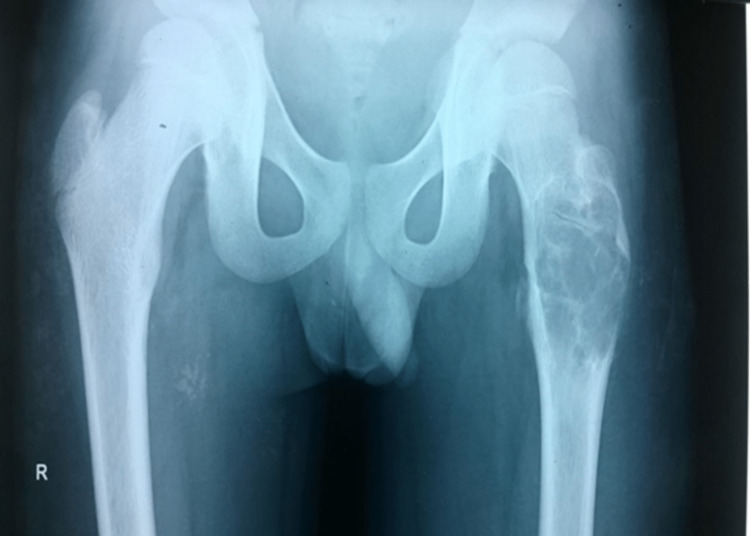
Lytic lesion in the left proximal femur (first visit)

A 17-year male patient presented to us with dull aching pain in the left hip for one year. X-ray showed a lytic lesion in the PF suggestive of a benign bone tumor (Figure 1).

**Figure 2:**
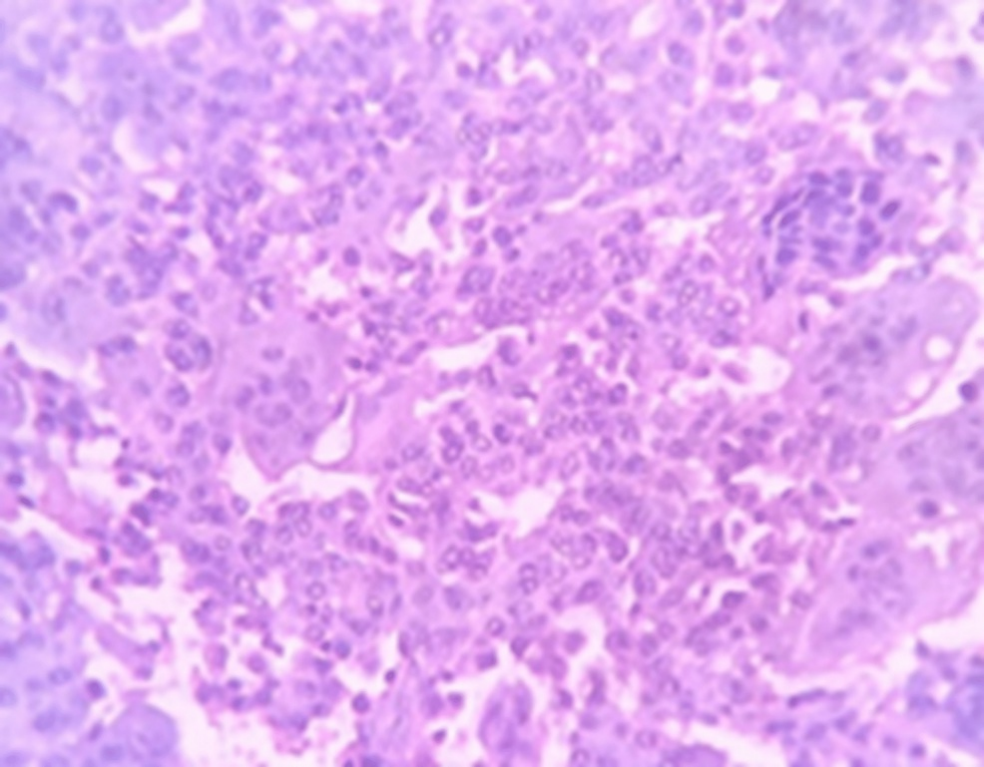
HPE suggestive of GCT HPE: Histopathological examination; GCT: Giant cell tumor.

Core needle biopsy was suggestive of GCTB (Figure 2).

**Figure 3:**
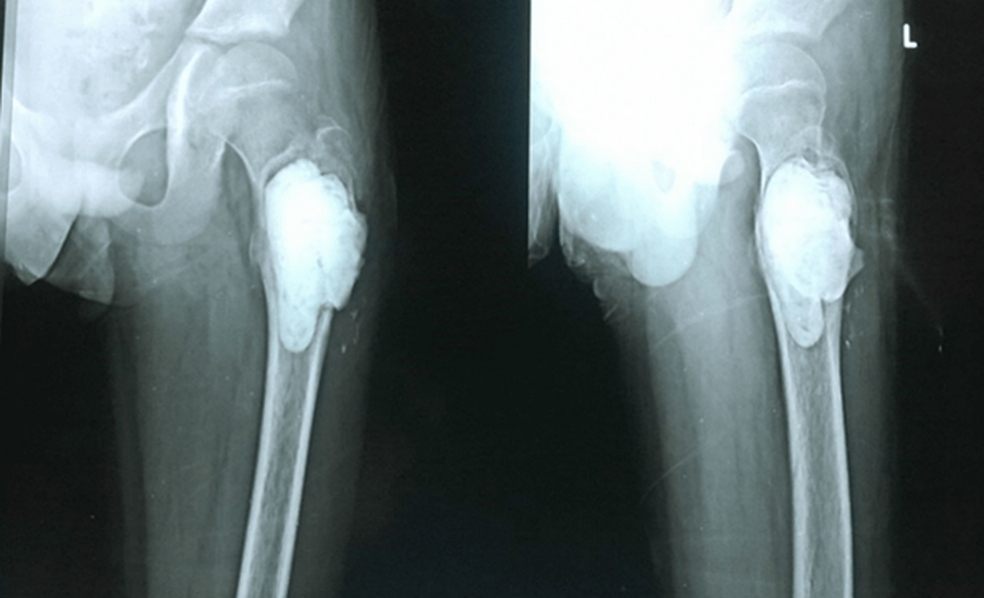
Curettage and cement augmentation

He underwent curettage, the use of phenol as an adjuvant, and vancomycin-coated antibiotic cement augmentation (Figure 3).

**Figure 4:**
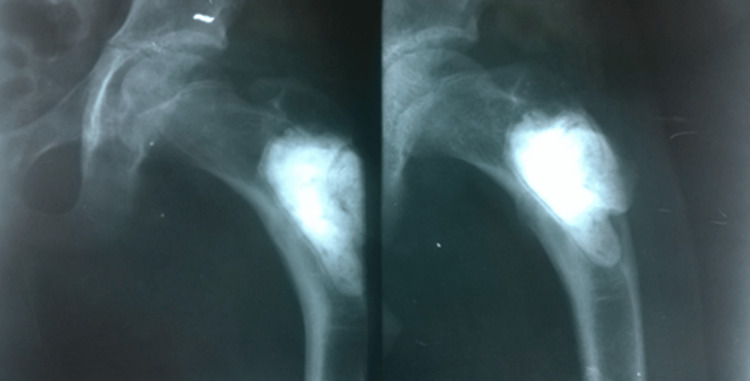
Development of coxa vara deformity after two years

The HPE report of the curetted material confirmed the lesion to be GCTB. Subsequently, he remained asymptomatic for two years. After two years, the patient started having a painful limp. An X-ray revealed a coxa vara deformity, which was then managed conservatively (Figure 4).

**Figure 5:**
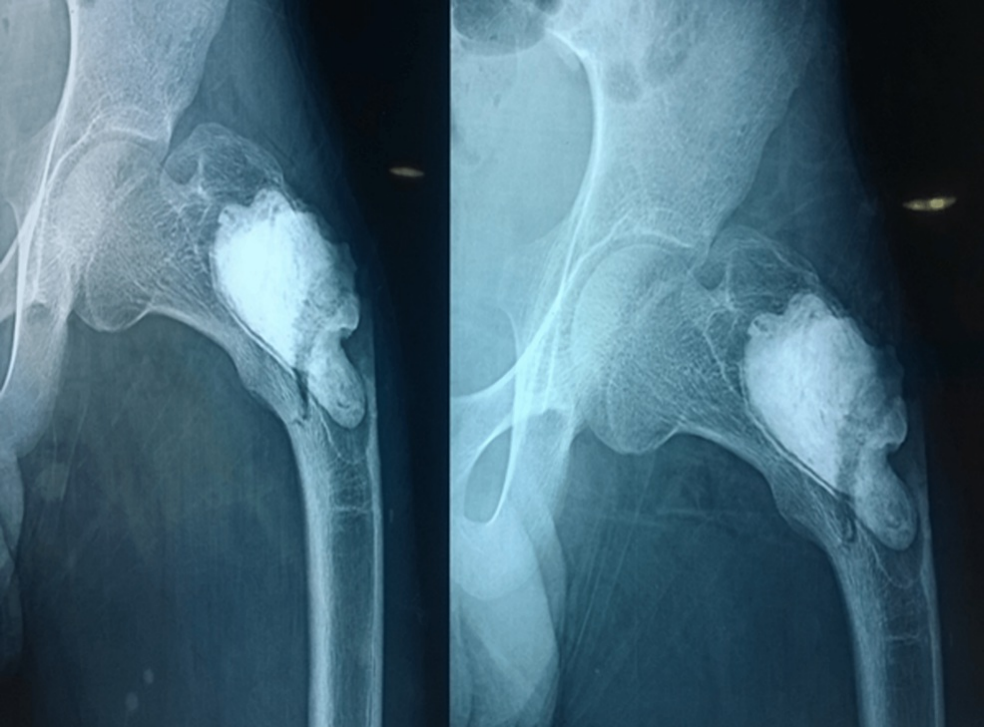
Subsequent fatigue fracture in the subtrochanteric region

He continued to walk with a short-limb gait, but after one month, he sustained a fatigue fracture in the subtrochanteric region (Figure 5).

**Figure 6:**
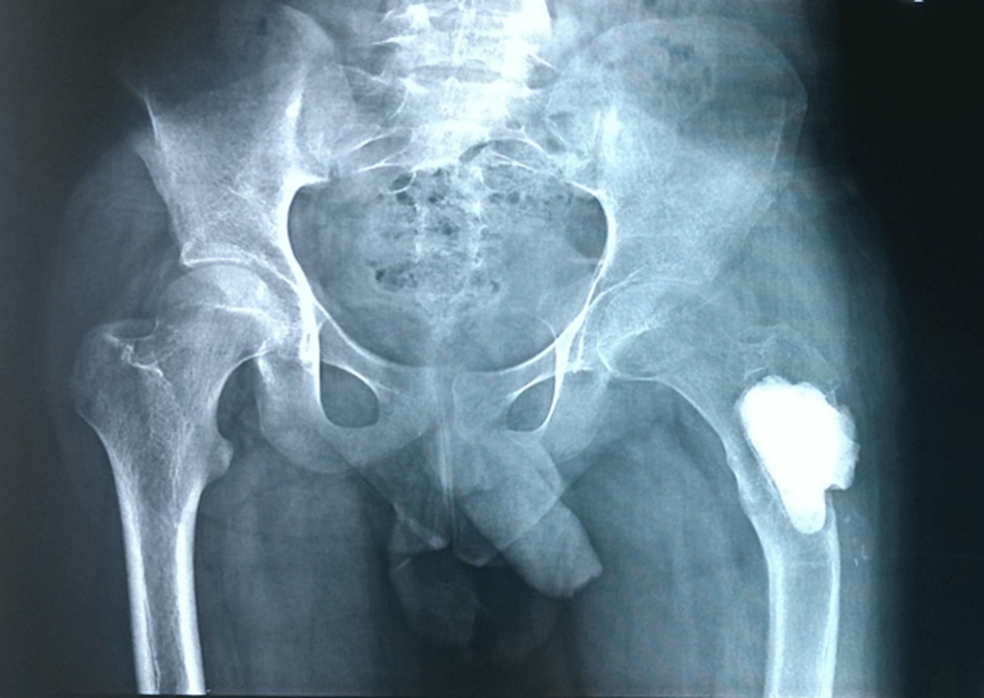
One-year follow-up following conservative management of subtrochanteric fatigue fracture

Following this, the patient was admitted and managed conservatively with skin traction under continuous monitoring for three months, which resulted in fracture union and improvement in the neck-shaft angle, after which the patient was gradually rehabilitated to full weight bearing. At the one-year follow-up subsequent to the initiation of conservative management of fracture, the X-ray showed complete union and normal neck-shaft angle (Figure 6).

**Figure 7:**
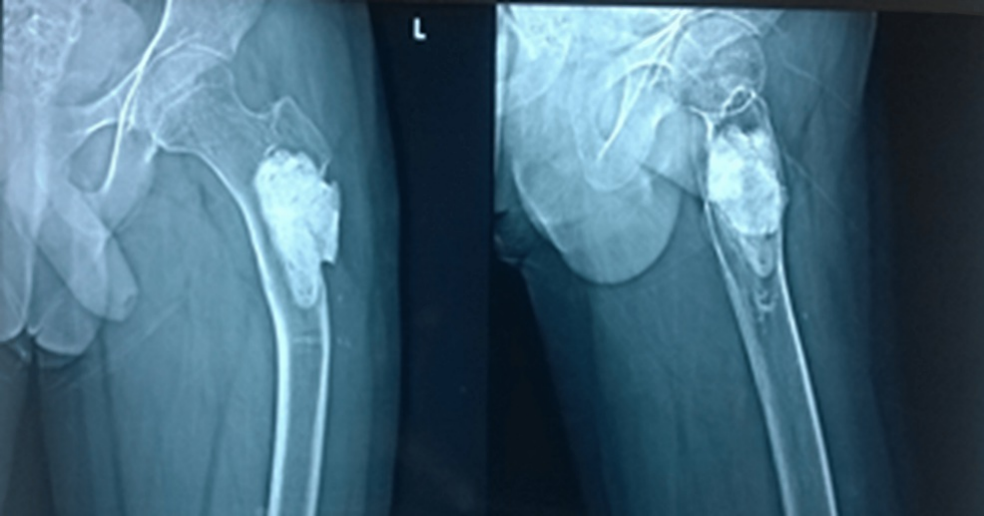
Redevelopment of coxa vara four years post index surgery

**Figure 8:**
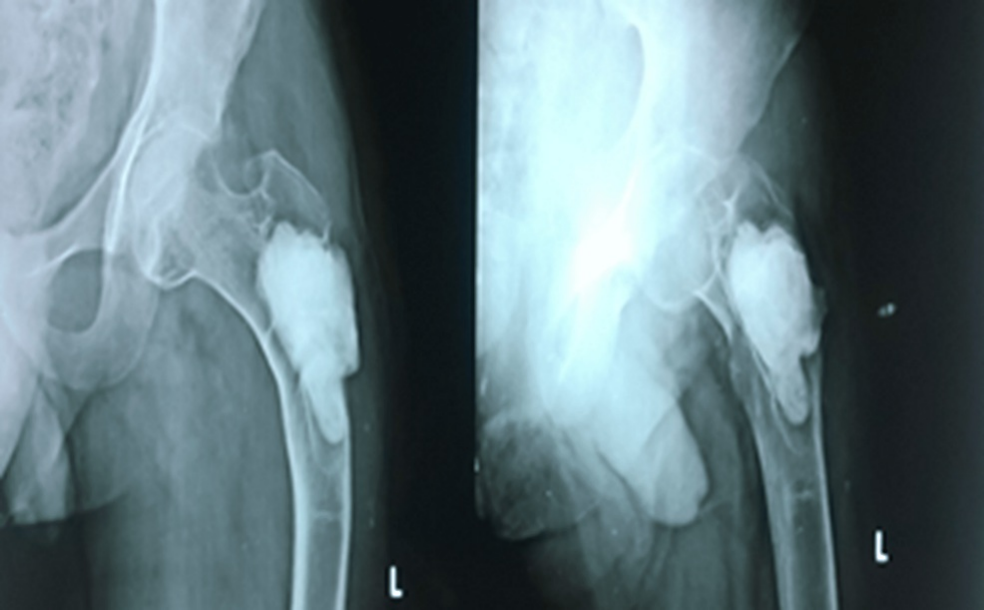
Re-fracture in the subtrochanteric region

However, after two years (four years post-index surgery), the patient redeveloped coxa vara deformity (Figure 7) with an evident subtrochanteric fracture three months later (Figure 8).

**Figure 9:**
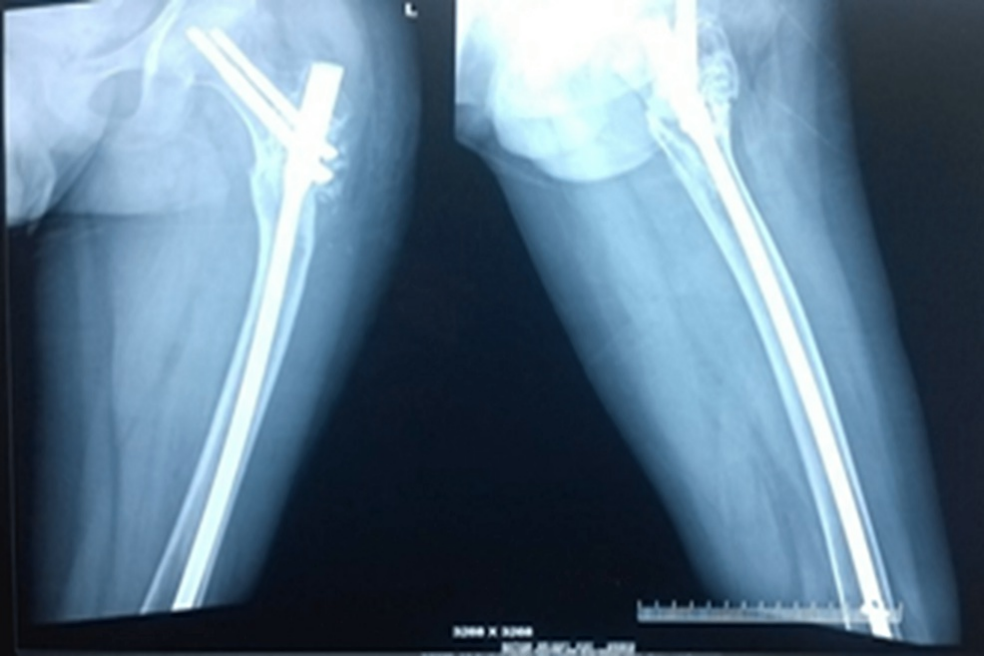
Removal of bone cement, IC, and IF with PFN + morselized autofibular bone grafting IC: Intralesional curettage; IF: Internal fixation; PFN: Proximal femoral nail.

He underwent removal of bone cement, intralesional curettage, internal fixation with PFN, and morselized autofibular bone grafting (Figure 9).

**Figure 10:**
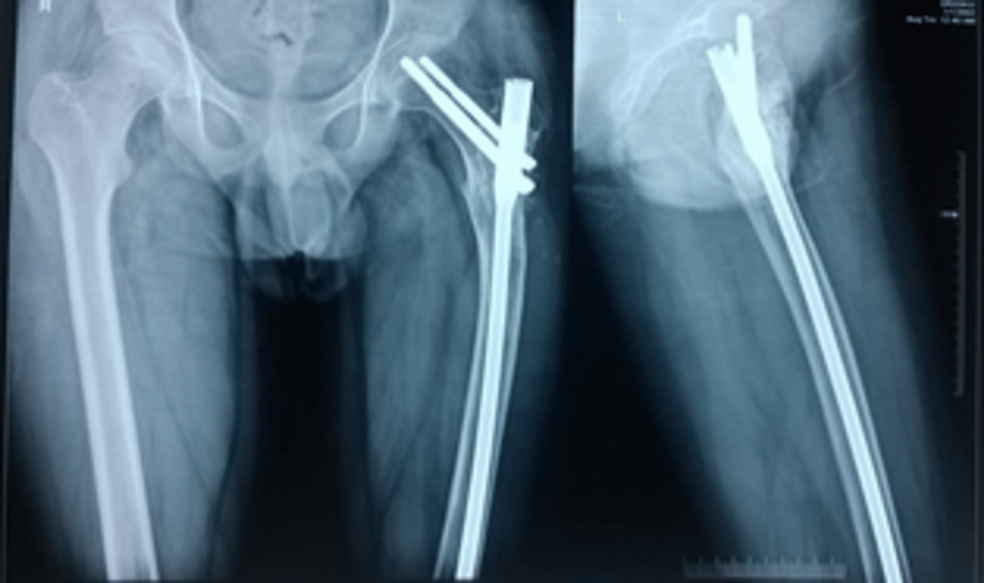
Two-year follow-up showing no signs of recurrence

Biopsy confirmed a multinucleated giant cell suggesting a recurrence of GCT in the PF. He was started with bisphosphonates (Tab Alendronate 70 mg weekly once) along with calcium and vitamin D3 supplements postoperatively for two years. The two-year follow-up showed fracture union without any signs of recurrence (Figure 10).

## Discussion

GCTB poses several challenges in its management. There are a number of complications that can be encountered while surgically managing a GCTB case. The most common complications are tumor recurrence, failed reconstruction, and postoperative fracture. The lack of conclusive assessment criteria for postoperative fracture risk and appropriate implants for augmentation/construct reinforcement are concerns that need to be addressed.

This article reviews the various factors responsible for postoperative fracture after curettage in GCT. Marcove et al. stated that curettage combined with cementation bears an increased fracture risk because of the premature load bearing of the involved bone as well as inadequate cement strength in the bone defect after curettage [7].

Role of defect size

According to Jeys et al. [8], a tumor volume to distal femoral volume ratio of 54% or greater was linked to a higher risk of pathological fracture. Amanatullah et al. showed an increased risk of fractures with defect sizes that damaged more than 50% of the cortical width in their biomechanical investigation [9]. On the distal femur, finite element calculations revealed that the bone strength dropped when the defect represented 35% or more of the epiphyseal volume and was greater in the medial defect than in the lateral defect [10,11].

Hirn et al. found that certain lesions were never fully filled up with new bone, while others were. They concluded that a key element in the development of postoperative fracture is the size of the defect. They claimed that when a bone defect's volume was higher than 60 cm^3^ and its maximal diameter was greater than 5 cm, the risk of fracture increased dramatically.

Role of adjuvants

Phenol has a lower fracture rate than liquid nitrogen because it penetrates the bone less extensively [12,13]. Infection, postoperative fracture or femoral condyle collapse, nonunion, nerve palsy, and PMMA leakage were the more common non-oncological problems found by van der Heijden et al. when liquid nitrogen was used by direct pour method [3]. The average depth of necrosis, according to Bombardier et al., was 2.54 mm for the liquid nitrogen spray and 0.3 mm for the phenol [14]. As a result, cryosurgery utilizing liquid nitrogen spray may maintain bone union ability. Cryosurgery using liquid nitrogen spray has been shown to lower fracture rates when compared to the direct pour technique (0% versus 17%) [15].

The majority of authors concur that high-speed burring of the cavity's borders is advantageous for sufficient exposure, but in some cases, especially when the bone is already thin, this may increase the risk of fracture [16].

Bone grafting versus bone cement

The defect once created after curettage of the tumorous area causes the host bone to weaken structurally. There are various advantages and disadvantages of using bone graft/cement found in the literature. However, there is no common consensus regarding the choice of material to be used for filling the bone defect created after curettage.

As cement has a higher Young's modulus than trabecular bone, reconstruction by bone cement leads to the transfer of load to the stiffer material causing resorption at the bone cement interface [17]. As a result, it weakens the nearby trabecular bone, which leads to fracture. This effect is more extensive near the joint where extensive curettage and cementation are done.

Benevenia et al. [2] reported that the cement with bone graft groups (5%) have a low risk of postoperative periarticular fracture compared to the cement group (23%). Wallace et al. discovered a statistically insignificant increase in postoperative fracture in patients who underwent bone graft reconstruction [18].

Role of implants in reconstruction

Construct reinforcement after curettage and cementation offers a mechanical advantage in reducing postoperative fracture. However, there is a paucity of literature that can guide in choosing implants. Large cavitary lesions are often supported by pins, screws, and locking plates as they provide immediate stability and structural support [19-23].

Various in vitro studies and finite element analyses on the bone model have supported the need for construct reinforcement with internal devices in larger cavitary lesions and concluded the superiority of nails/plates over pins and screws as they span the proximal and distal end of bone cement interface and their screws cross the interior wall of the interface [24,25].

There is a scarcity of literature on stabilizing a PF GCT with intramedullary fixation following adequate curettage. Even though four of the 10 patients in his group who received this type of treatment experienced local recurrences, Sakayama et al. advised against replacement if at all possible [26].

Role of bisphosphonates and denosumab

By making the bone lesions harden, the monoclonal antibody denosumab aids in halting the breakdown of the bone. It works by attaching to the nuclear factor-kappa ligand's active receptor. According to studies, tumor cells are concealed in osteosclerotic lesions, and keeping them untreated increases the risk of recurrence [27,28]. Every four weeks, 120 mg of denosumab can be injected subcutaneously, with an extra 120 mg loading dosage given on days 8 and 15 of the first cycle. It can be continued until either radiographic disease stability is achieved at six months [29] or there is nearly complete eradication of large cells on repeat biopsies following treatment (for all evaluable patients) [30]. Due to denosumab's tumoristatic properties, stromal cells in the sclerotic rim may take longer to reactivate, increasing the likelihood of recurrence [31].

On osteoclasts, which are precursors of GCTB, zoledronic acid has an apoptotic impact. A consensus has not been reached because there is conflicting evidence supporting the use of zoledronic acid in GCTB prevention. Supplementing with zoledronic acid has been linked to noticeably decreased rates of tumor recurrence [32]. Preoperatively, three doses of intravenous zoledronic acid (4 mg) were administered in GCTB at intervals of three weeks in a trial by Zile et al. Two weeks following the last infusion, the extended curettage was carried out. Recurrence only happened once among the 18 patients in the infusion group, while it happened four times among the 19 patients in the control group [33].

Bisphosphonates such as oral alendronate have been shown to have similar effectiveness in reducing the recurrence of GCTB [34]. Alendronate can be given orally to patients with GCTB who have undergone surgery in the dosage of 70 mg/week or 10 mg once every day [35]. It can be given for a period of two to three years. It has been shown to prevent recurrence along with fewer side effects when compared to zoledronic acid. Other bisphosphonates such as ibandronate have also been used and have been found effective in preventing the recurrence of GCTB [36].

There is a need for assessment criteria that can guide the surgical approach in GCT. Chinese doctors devised a grading system for surgical recommendations for treating GCTB that identified articular surface involvement, pathological fracture, and cortical bone degradation as risk factors [36].

In our study, we attempted to correct the coxa vara deformity after curettage and filled the cavity with a morselized fibular graft, and the construct was reinforced with an intramedullary device PFN. The follow-up of the patient has highlighted a promising result of the construct in GCT of the PF.

## Conclusions

Removal of tumor mass weakens the parent bone. This requires augmentation either with autologous/allogenic bone graft, bone graft substitutes, cement, or a combination of the two with or without instrumentation. Concerns that need to be addressed include the lack of clear assessment criteria for postoperative fracture risk and suitable implants for augmentation/construct reinforcement. Based on our experience regarding this case and available literature, we recommend instrumentation to prevent the deformity and help early healing of bone, when the cavity size is large and high stress area are involved.

## References

[1] Campanacci M, Baldini N, Boriani S, Sudanese A (1987). Giant-cell tumor of bone. J Bone Joint Surg Am.

[2] Benevenia J, Rivero SM, Moore J, Ippolito JA, Siegerman DA, Beebe KS, Patterson FR (2017). Supplemental bone grafting in giant cell tumor of the extremity reduces nononcologic complications. Clin Orthop Relat Res.

[3] van der Heijden L, Dijkstra PD, Campanacci DA, Gibbons CL, van de Sande MA (2013). Giant cell tumor with pathologic fracture: should we curette or resect?. Clin Orthop Relat Res.

[4] van der Heijden L, van der Geest IC, Schreuder HW, van de Sande MA, Dijkstra PD (2014). Liquid nitrogen or phenolization for giant cell tumor of bone?: a comparative cohort study of various standard treatments at two tertiary referral centers. J Bone Joint Surg Am.

[5] Dahlin DC, Unni KK (1986). Bone Tumors: General Aspects and Data on 8,547 Cases. https://www.osti.gov/biblio/5518830.

[6] Wijsbek AE, Vazquez-Garcia BL, Grimer RJ, Carter SR, Abudu AA, Tillman RM, Jeys L (2014). Giant cell tumour of the proximal femur: is joint-sparing management ever successful?. Bone Joint J.

[7] Marcove RC, Weis LD, Vaghaiwalla MR, Pearson R (1978). Cryosurgery in the treatment of giant cell tumors of bone: a report of 52 consecutive cases. Clin Orthop Relat Res.

[8] Jeys LM, Suneja R, Chami G, Grimer RJ, Carter SR, Tillman RM (2006). Impending fractures in giant cell tumours of the distal femur: incidence and outcome. Int Orthop.

[9] Amanatullah DF, Williams JC, Fyhrie DP, Tamurian RM (2014). Torsional properties of distal femoral cortical defects. Orthopedics.

[10] Ghouchani A, Rouhi G, Ebrahimzadeh MH (2020). Post-operative fracture risk assessment following tumor curettage in the distal femur: a hybrid in vitro and in silico biomechanical approach. Sci Rep.

[11] Ghouchani A, Rouhi G, Ebrahimzadeh MH (2019). Investigation on distal femoral strength and reconstruction failure following curettage and cementation: in-vitro tests with finite element analyses. Comput Biol Med.

[12] Mittag F, Leichtle C, Kieckbusch I, Wolburg H, Rudert M, Kluba T, Leichtle U (2013). Cytotoxic effect and tissue penetration of phenol for adjuvant treatment of giant cell tumours. Oncol Lett.

[13] Jacobs PA, Clemency RE Jr (1985). The closed cryosurgical treatment of giant cell tumor. Clin Orthop Relat Res.

[14] Bombardier B, Haase D, Sweeney K, Friedman E, Poppe T, Hughes N (2021). A comparison of depth of necrosis among adjuvant therapies used for the treatment of benign bone tumors. J Surg Oncol.

[15] Lee RJ, Mayerson JL, Crist M (2011). Fracture risk with pressurized-spray cryosurgery. Am J Orthop (Belle Mead NJ).

[16] Khan SA, Kumar A, Inna P, Bakhshi S, Rastogi S (2009). Endoprosthetic replacement for giant cell tumour of the proximal femur. J Orthop Surg (Hong Kong).

[17] Morley T (2008). Vertebral tumors. J Bone Joint Surg Br.

[18] Wallace MT, Henshaw RM (2014). Results of cement versus bone graft reconstruction after intralesional curettage of bone tumors in the skeletally immature patient. J Pediatr Orthop.

[19] Li J, Wodajo F (2013). Computer simulation techniques in giant cell tumor curettage and defect reconstruction. Comput Sci Eng.

[20] Murray PJ, Damron TA, Green JK, Morgan HD, Werner FW (2004). Contained femoral defects: biomechanical analysis of pin augmentation in cement. Clin Orthop Relat Res.

[21] Toy PC, Arthur S, Brown D, Heck RK (2007). Reconstruction of noncontained proximal tibial defects with divergent screws and cement. Clin Orthop Relat Res.

[22] Uglialoro AD, Maceroli M, Beebe KS, Benevenia J, Patterson FR (2009). Distal femur defects reconstructed with polymethylmethacrylate and internal fixation devices: a biomechanical study. Orthopedics.

[23] Toy PC, France J, Randall RL, Neel MD, Shorr RI, Heck RK (2006). Reconstruction of noncontained distal femoral defects with polymethylmethacrylate and crossed-screw augmentation: a biomechanical study. J Bone Joint Surg Am.

[24] Ahmadi S, Shah S, Wunder JS, Schemitsch EH, Ferguson PC, Zdero R (2013). The biomechanics of three different fracture fixation implants for distal femur repair in the presence of a tumor-like defect. Proc Inst Mech Eng H.

[25] Ruskin J, Caravaggi P, Beebe KS (2016). Steinmann pin augmentation versus locking plate constructs. J Orthop Traumatol.

[26] Sakayama K, Sugawara Y, Kidani T (2007). Diagnostic and therapeutic problems of giant cell tumor in the proximal femur. Arch Orthop Trauma Surg.

[27] Errani C, Tsukamoto S, Leone G, Righi A, Akahane M, Tanaka Y, Donati DM (2018). Denosumab may increase the risk of local recurrence in patients with giant-cell tumor of bone treated with curettage. J Bone Joint Surg Am.

[28] Tsukamoto S, Mavrogenis AF, Akahane M (2022). Risk factors of fracture following curettage for bone giant cell tumors of the extremities. BMC Musculoskelet Disord.

[29] Xu SF, Adams B, Yu XC, Xu M (2013). Denosumab and giant cell tumour of bone-a review and future management considerations. Curr Oncol.

[30] Kanwat H, Banjara R, Kumar VS, Majeed A, Gamnagatti S, Khan SA (2021). Comparison of denosumab and zoledronic acid as neoadjuvant therapy in patients with giant cell tumor of bone. J Orthop Surg (Hong Kong).

[31] Kumar A, Sinha S, Haider Y, Jameel J, Kumar S (2021). Role of zoledronic acid supplementation in reducing post-surgical recurrence of giant cell tumor of bone: a meta-analysis of comparative studies. Cureus.

[32] Kundu ZS, Sen R, Dhiman A, Sharma P, Siwach R, Rana P (2018). Effect of intravenous zoledronic acid on histopathology and recurrence after extended curettage in giant cell tumors of bone: a comparative prospective study. Indian J Orthop.

[33] Shi M, Chen L, Wang Y, Wang W, Zhang Y, Yan S (2019). Effect of bisphosphonates on local recurrence of giant cell tumor of bone: a meta-analysis. Cancer Manag Res.

[34] Pannu CD, Kandhwal P, Raghavan V, Khan SA, Rastogi S, Jayaswal A (2018). Role of bisphosphonates as adjuvants of surgery in giant cell tumor of spine. Int J Spine Surg.

[35] Zhang W, Zhang Y, Li P (2011). Administration of sodium ibandronate in the treatment of complicated giant cell tumor of the spine. Spine (Phila Pa 1976).

[36] Lun DX, Hu YC, Yang XG (2018). Development and proposal of a scoring system for giant cell tumour of the bone around the knee. Int Orthop.

